# Supplementary material for: Identification of photoperiod-induced specific miRNAs in the adrenal glands of Sunite sheep (Ovis aries)
Source: Front Vet Sci. 2022 Jul 22;9:888207. doi: 10.3389/fvets.2022.888207 (PMC9354845; doi:10.3389/fvets.2022.888207)
Supplement: Supplementary file 1 [file Data_Sheet_1.ZIP › Supplementary file/miRDeep2 file.zip!/miRDeep2 file/Novel_Structure/SP42A2/Novel_599.pdf]

Provisional ID	:	NC_019484.2_42694
Score total	:	2.5
Score for star read(s)	:	3.9
Score for read counts	:	-3.9
Score for mfe	:	0.9
Score for randfold	:	1.6
Score for cons. seed	:	
Total read count	:	4
Mature read count	:	2
Loop read count	:	0
Star read count	:	2

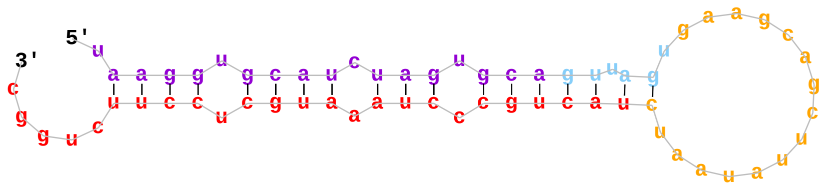

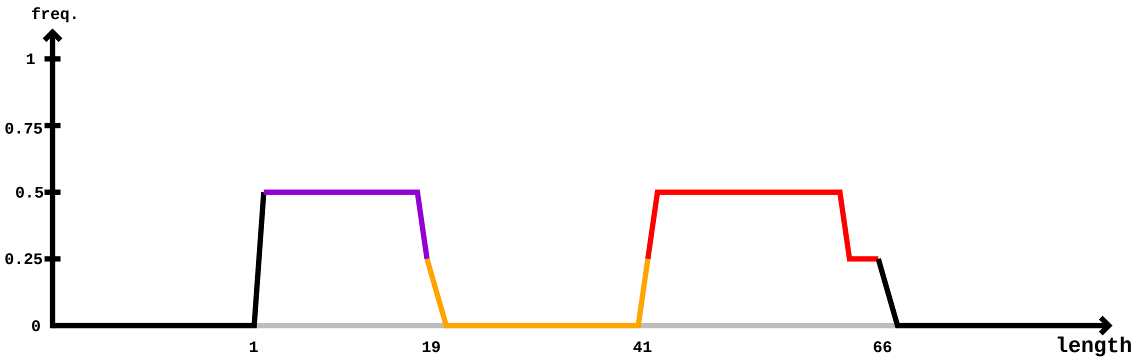


```
                        Star                                     Mature
5'- ccauaaugugucucuuguguuaaggugcaucuagugcaguuagugaagcagcuuauaaaucuacugcccuaaaugcuccuucuggcacaggcugccugacacacagcau  -3'  obs
    ccauaaugugucucuuguguuaaggugcaucuagugcaguuagugaagcagcuuauaaaucuacugcccuaaaugcuccuucuggcacaggcugccugacacacagcau       exp
    ......((((((.((((((((((((.((((.(((.(((((.((.................))))))).))).)))).)))..)))))))))......))))))......  reads  mm  sample
    ....................uaaggugcaucuagugc........................................................................  1      0   seq
    ....................uaaggugcaucuagugca.......................................................................  1      0   seq
    ............................................................uacugcccuaaaugcuccuucuggc........................  1      0   seq
    .............................................................acugcccuaaaugcuccuuc............................  1      0   seq
```
